# Preparation of Expanded Graphite-VO_2_ Composite Cathode Material and Performance in Aqueous Zinc-Ion Batteries

**DOI:** 10.3390/ma17122817

**Published:** 2024-06-10

**Authors:** Jiaye Li, Jing Zhao, Zebin Wang, Huan Liu, Qing Wen, Jinling Yin, Guiling Wang

**Affiliations:** 1 Key Laboratory of Superlight Materials and Surface Technology of Ministry of Education, College of Materials Science and Chemical Engineering, Harbin Engineering University, Harbin 150001, China; 18804627205@163.com (J.L.); wangzb123@hrbeu.edu.cn (Z.W.); huan9946521@163.com (H.L.); wenqing@hrbeu.edu.cn (Q.W.); yinjinling@hrbeu.edu.cn (J.Y.); 2 Heilongjiang Hachuan Carbon Materials Technology Co., Ltd., National Quality Supervision and Inspection Center of Graphite Products, No. 88 Kangxin Road, Jiguan District, Jixi 158100, China

**Keywords:** aqueous zinc-ion battery, cathode materials, carbon composite

## Abstract

Due to safety problems caused by the use of organic electrolytes in lithium-ion batteries and the high production cost brought by the limited lithium resources, water-based zinc-ion batteries have become a new research focus in the field of energy storage due to their low production cost, safety, efficiency, and environmental friendliness. This paper focused on vanadium dioxide and expanded graphite (EG) composite cathode materials. Given the cycling problem caused by the structural fragility of vanadium dioxide in zinc-ion batteries, the feasibility of preparing a new composite material is explored. The EG/VO_2_ composites were prepared by a simple hydrothermal method, and compared with the aqueous zinc-ion batteries assembled with a single type of VO_2_ under the same conditions, the electrode materials composited with high-purity sulfur-free expanded graphite showed more excellent capacity, cycling performance, and multiplicity performance, and the EG/VO_2_ composites possessed a high discharge ratio of 345 mAh g^−1^ at 0.1 A g^−1^, and the Coulombic efficiency was close to 100%. The EG/VO_2_ composite has a high specific discharge capacity of 345 mAh g^−1^ at 0.1 A g^−1^ with a Coulombic efficiency close to 100%, a capacity retention of 77% after 100 cycles, and 277.8 mAh g^−1^ with a capacity retention of 78% at a 20-fold increase in current density. The long cycle test data demonstrated that the composite with expanded graphite effectively improved the cycling performance of vanadium-based materials, and the composite maintained a stable Coulombic efficiency of 100% at a high current density of 2 A/g and still maintained a specific capacity of 108.9 mAh/g after 2000 cycles.

## 1. Introduction

In recent decades, the growing demand for clean energy has triggered the rapid commercialization of renewable energy technologies (e.g., solar, wind, tidal, etc.) due to concerns about limited fossil fuel resources and environmental degradation [1,2]. Electrochemical batteries have the advantages of high energy/power density, fast response, and long cycle life [3]. Lithium-ion batteries, the most widely used electrochemical batteries today, are not the first choice because they require the use of flammable organic electrolytes and expensive electrode materials, which make them face severe operational safety and cost issues for large-scale renewable energy storage. As a potential alternative, aqueous batteries are ideal for energy storage applications due to their unique advantages such as low cost, high safety, easy processing, environmental friendliness, and high ionic conductivity [4]. Among the various cationic aqueous batteries studied so far, zinc-ion batteries with Zn as the negative electrode stand out. Its higher theoretical capacity, air stability, water stability, soil abundance, and non-toxicity make it unique among aqueous batteries in meeting capacity, cost, and safety requirements [5].

Manganese dioxide-based cathode materials [6,7,8,9], Prussian blue cathode materials [10,11,12,13], and vanadium pentoxide-related materials [14,15] are currently receiving a lot of attention and have been much discussed. However, their development has been limited by the poor cycling stability of manganese dioxide cathodes and the inherently low capacity of Prussian blue analogs. The various valence states (from +2 to +5) of the element vanadium (V) allow vanadium-based materials to have different crystal structures and electrochemical properties [16,17]. For instance, V_2_O_5_ [18,19,20,21], VO_2_ [22,23], V_2_O_3_ [24], V_3_O_7_ [25], and V_6_O_13_ [14] are highly promising materials for aqueous zinc-ion batteries. However, the fragile structure [26] as well as the slightly water-soluble characteristics [27,28] affect the stability of VO_2_ material, which restricts the process of its practical application, and thus it has become a promising electrode material that has been paid little attention in the study of aqueous zinc-ion cathode materials. In recent years, researchers around the world have been working to improve the electrochemical performance of VO_2_ electrode materials. The following strategies [29,30] may be effective: (1) Composite with highly conductive materials. (2) Increasing the layer spacing of cathode materials to provide enough diffusion space for Zn^2+^. We propose a hydrothermal composite of expanded graphite and vanadium dioxide to construct a cathode material that combines performance and stability by taking advantage of the electrical conductivity and stability of expanded graphite, and we investigate the electrochemical properties of the composite material through a series of characterization methods and electrochemical tests. We proposed a hydrothermal composite of expanded graphite and vanadium dioxide to construct a cathode material that combines performance and stability by taking advantage of the electrical conductivity and stability of expanded graphite, and we investigated the electrochemical properties of the composite material through a series of electrochemical tests.

## 2. Materials and Methods

### 2.1. Raw Materials

The raw materials of flake graphite (Carbon Content > 99.95%) were purchased from CNBM Heilongjiang Graphite New Materials Co., Ltd., Heilongjiang, China, and the zinc sulfate heptahydrate (ZnSO_4_·7H_2_O), vanadium pentoxide (V_2_O_5_), and citric acid monohydrate (C_6_H_8_O_7_·H_2_O) were analytically pure and purchased from Sinopharm Chemical Reagent Co., Shanghai, China. The battery assembly components used in this paper are battery grade and purchased from Guangdong Candlelight New Energy Co., Guangdong, China.

### 2.2. Preparation of Composite Cathode Materials

We added 3 mmol of vanadium pentoxide into 30 mL deionized water and heated and stirred the mixture until fully dissolved. Then, 3 mmol of expanded graphite material was added into the solution and continued to stir until the expanded graphite was uniformly dispersed in the system. Finally, 5 mmol of citric acid was added into the above solution and continued to stir for 30 min. The stirred solution was then ultrasonicated for 30 min to assist the raw material in entering the expanded graphite interlayer. Subsequently, the homogeneous solution was transferred to a Teflon hydrothermal reactor and subjected to a hydrothermal reaction at 180 °C for 12 h. After the completion of the reaction, the prepared samples were rinsed and filtered three times with deionized water and anhydrous ethanol. The black powder obtained after centrifugation and drying was identified as EG/VO_2_. The process of preparing vanadium dioxide was the same as the above steps, except that expanded graphite is not added to the preparation process. Firstly, the active material of the electrode, the conductive carbon black, and the binder PVDF were mixed in a small crucible at a ratio of 8:1:1, and then an appropriate amount of N-methyl pyrrolidone was added as a solvent. After stirring in the crucible for 4–6 h to form a homogeneous electrode slurry, it was scraped and coated on the titanium foil, and then the coated titanium foil was put into a vacuum drying oven and dried at 60 degrees Celsius for 12 h. After that, it was taken out and then it was prepared as the positive electrode of the aqueous zinc-ion battery. The negative electrode was constructed using zinc foil with a thickness of 0.02 mm. Before use, the zinc foil should be polished with sandpaper to create a bright mirror surface, any the dirt on the surface of the zinc foil should be removed, and then it is immersed into anhydrous ethanol solution and ultrasonically cleaned for 30 min.

### 2.3. Characterization and Testing Methods

The scanning electron microscope model JSM-6480, manufactured by Nippon Electron Corporation (Tokyo, Japan), and the high-resolution projection electron microscope model TALOS G2 F200X manufactured by FEI Corporation (Eindhoven, The Netherlands) were used to observe the microscopic morphology of the samples [31,32,33]. The X-ray photoelectron spectrometer model ESCALAB 250 from Thermo Fisher Scientific, Waltham, MA, USA, was used to determine the elemental composition, valence, and chemical bonding of the material surface [34]. The model HR800 Raman spectrometer produced by Jobin Yvon (Paris, France) was used for Raman spectroscopy. The fully automatic gas adsorption meter, model ASAP29200, produced by Quantachrome, Boynton Beach, FL, USA, was used for BET analysis [35,36].

## 3. Results

### 3.1. Characterization

**Figure 1:**
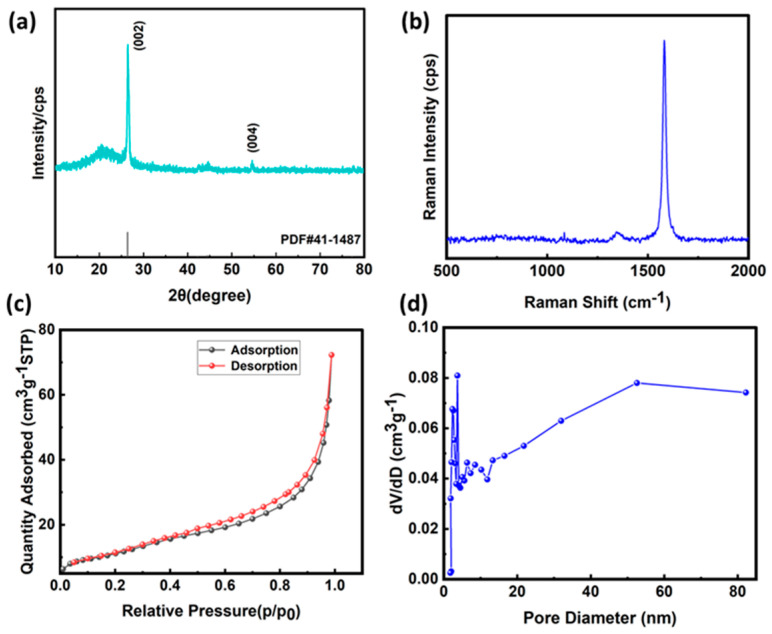
(**a**) XRD spectrum of expanded graphite. (**b**) Raman spectrum of expanded graphite. (**c**) Nitrogen absorption/desorption isotherm of EG. (**d**) Pore size distribution of EG.

As shown in Figure 1a, two characteristic peaks were observed in the expanded graphite at 2θ = 26.44° and 2θ = 54.54°, which corresponded to the (002) crystalline surface and (004) crystalline surface of the graphite material, and the positions of these peaks were basically the same as that of the natural scaled graphite compared to the graphite material, but its strength is greatly reduced, which is caused by the decomposition of the intercalation material and the gasification of the graphite material by the high temperature. The reason is that in the process of preparing expanded graphite by high temperature, the interpolated material decomposes and gasifies, which pushes the graphite carbon layer to expand and distort along the C-axis, which destroys the original crystal structure of natural flake graphite and further reduces its crystallinity, resulting in the reduction of the intensity of the diffraction peak. But the expanded graphite still retains the crystal structure of natural flake graphite to a certain extent. The Raman spectrum (532 nm) is shown in Figure 1b, which shows two typical peaks located at 1350 cm^−1^ and 1580 cm^−1^, which correspond to the D and G bands of the expanded graphite, respectively. The spectral analysis of EG showed that the typical G-band peak appeared at 1580 cm^−1^, which was caused by the stretching vibration of all sp2 atom pairs in the carbon ring or long chain. The D-band peak appeared at 1350 cm^−1^, which was caused by the defects and disorder induced in the graphite. The specific surface area of the expanded graphite was determined by a specific surface area analyzer using the nitrogen suction-desorption method, and the nitrogen suction-desorption curves and pore size distribution are shown in Figure 1c,d. From Figure 1c, it can be seen that the type of adsorption isotherm belongs to type V, indicating its classification as a hydrophobic macroporous material. Additionally, the pore size distribution graph (Figure 1d) reveals that the pore size distribution of expanded graphite is dominated by small pores, and a certain amount of large pores also exist. The specific surface area of the expanded graphite is 40.83 m^2^/g, which indicates that the prepared expanded graphite has a large specific surface area.

**Figure 2:**
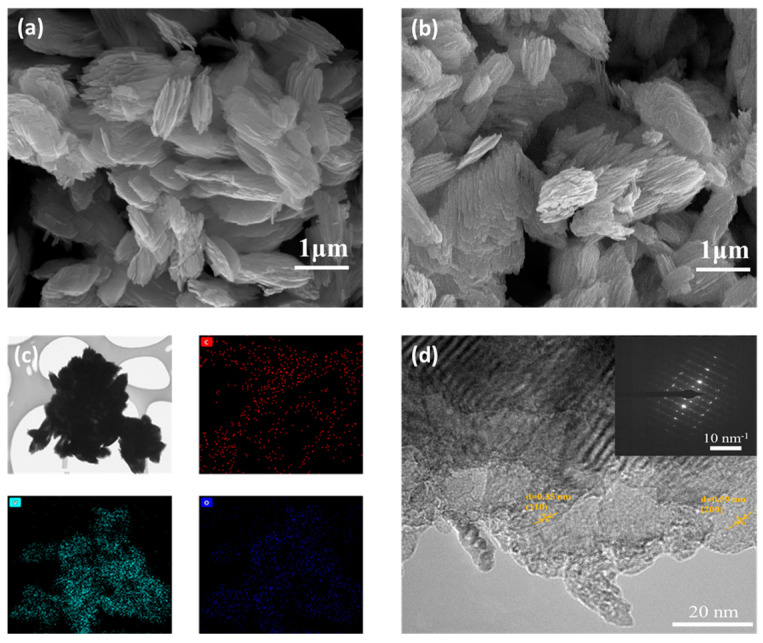
(**a**) SEM image of the VO_2_ nanomaterial, (**b**) SEM image of the EG/VO_2_ composite, (**c**) TEM image with EDS mapping of the EG/VO_2_ composite, (**d**) HR-TEM image of the EG/VO_2_ composite.

Figure 2a,b show the micro-morphological features of pure VO_2_ and EG/VO_2_ composites, and the surface of the expanded graphite is covered by layers of stacked and folded nanosheets. There exists a distinct gap between these nanosheets, and this specific structure endows the cathode material with more active sites, and at the same time makes the material flexible so that it is not easy to be destroyed during charging and discharging. The thickness of the vanadium oxide nanomaterials is about 40 nm, and the length and width are in the range of 200~300 nm. There is no significant change in the particle size of VO_2_ with the addition of expanded graphite to the system for the preparation of VO_2_ nanomaterials, indicating that the deposition of VO_2_ on the surface of EG does not affect its crystallization.

Figure 2c shows the TEM and EDS images of the composites, and the transmission image reveals that the VO_2_ exhibits a shuttle-shaped lamellar stacking morphology and it is confirmed by the EDS-Mapping technique that proves that the elements C, V, and O are uniformly distributed on the expanded graphite in the EG/VO_2_ samples, and no other elements are present, which further confirms that the VO_2_ successfully covered the EG surface. The lattice spacing of the nanomaterials is labeled in the HRTEM (Figure 2d) as 0.35 nm and 0.59 nm, which corresponds to (110) and (200) in the XRD spectra, respectively, and this proves that the vanadium oxide materials have been successfully prepared, which is consistent with the description of the related literature [36,37,38].

**Figure 3:**
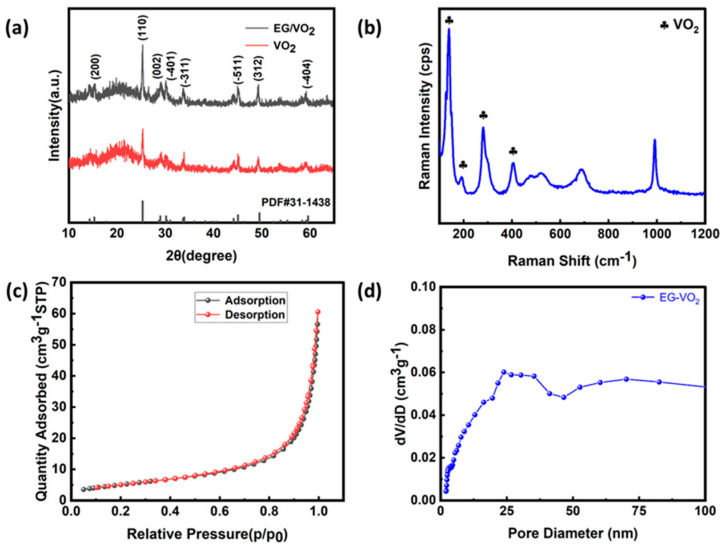
(**a**) XRD spectra of the EG/VO_2_ and VO_2_. (**b**) Raman spectra of EG/VO_2_. (**c**) Nitrogen absorption–desorption isotherms of EG/VO_2_. (**d**) Pore size distribution of EG/VO_2_.

X-ray diffraction (XRD) was used to characterize the crystal structures of the VO_2_ nanomaterial and EG/VO_2_ composite powders synthesized by hydrothermal method. As shown in Figure 3a, the three strong diffraction peaks of two materials located near 25°, 45°, and 49° correspond to the (110), (−511), and (312) crystal planes of the monoclinic crystal system of VO_2_(B) (PDF#31-1438), respectively, which is in agreement with the results of the other literature [39,40,41]. By XRD analysis, it is shown that VO_2_ is successfully compounded and the incorporation of expanded graphite did not affect the crystal structure of vanadium dioxide nanomaterials. The composition of the EG/VO_2_ composites was further confirmed by Raman tests, and the results are shown in Figure 3b. It can be observed that the peaks at 192, 223, 306, and 400 cm^−1^ in the spectra correspond to the characteristic peaks of vanadium dioxide, which belong to the Ag vibrational mode, which is related to the stretching motion of the V-V dimer. The sharp peaks near 689 cm^−1^ and 998 cm^−1^ may be due to the high laser intensity that led to the oxidation reaction of part of the vanadium dioxide, so the peaks were shifted.

In addition, the specific surface area and pore size distribution of the EG/VO_2_ samples were investigated and analyzed by BET nitrogen adsorption–desorption curves. The specific surface area of the EG/VO_2_ composite was calculated from the adsorption–desorption isotherm in Figure 3c to be 20.26 m^2^ g^−1^. This value is in agreement with the results of the nano-oxide material. The pore size distribution of the composites is shown in Figure 3d. The value of the pore size distribution of micropores is less than 2 nm, and if the pore size is between 2 and 10 nm, it means that there are more mesopores in the sample. It can be observed in the figure that the EG/VO_2_ material has more mesopores. These smaller pore sizes mainly originate from the nanoparticles themselves, which provide effective channels for ion transport, thus helping the electrolyte ions to enter the electrodes smoothly and thus optimizing the electrochemical performance of the composites. On the other hand, the larger pore size consists mainly of voids between nanoparticles and gaps between expanded graphite, and these large pores are capable of storing a large amount of electrolyte, which provides convenient conditions for the embedding and de-embedding of zinc ions. Therefore, the composites provide a high specific capacity for the battery through their unique pore structure.

**Figure 4:**
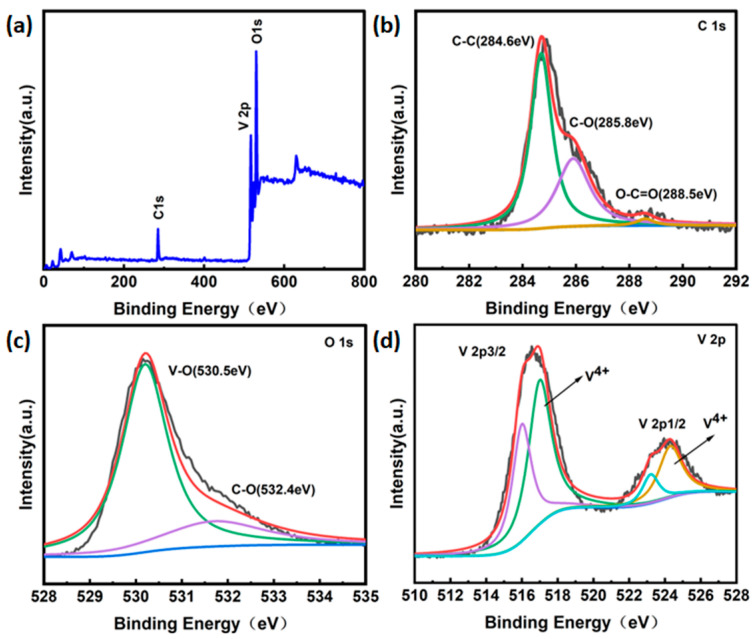
(**a**) The XPS of the full spectrum of the electrode material. (**b**) The XPS of the carbon element of the electrode material. (**c**) The XPS of the oxygen element of the electrode material. (**d**) The XPS of the vanadium element of the electrode material. The different colored lines in the Figure 4 (**b**–**d**) represent the fitted peaks for different chemical bonds.

To further analyze the elemental valence and chemical composition in the EG/VO_2_ composites, the samples were subjected to XPS tests. Figure 4a shows the full spectrum of the EG/VO_2_ composite, from which the characteristic peaks of C 1s, V 2p, and O 1s can be seen, indicating that the material contains three elements, C, V, and O. Figure 4b shows the splitting peaks of C 1s, and the three fitted peaks at 284.6 eV, 285.8 eV, and 288.5 eV of the binding energy correspond to C-C, C-O, and O-C=O bonds, respectively. Figure 4c shows that the O 1s XPS spectra are divided into two peaks, and the fitted peaks with binding energies of 530.5 eV and 532.4 eV belong to the V-O bond and the free oxygen element, respectively. The V 2p XPS spectra of EG/VO_2_ are shown in Figure 4d, the V 2p3/2 and V 2p1/2 diffraction peaks with binding energies of 516.5 eV and 523.5 eV correspond to V^3+^, and the V 2p3/2 and V 2p1/2 diffraction peaks at binding energies of 517.7 eV and 524.6 eV correspond to V^4+^. It is confirmed that both V^3+^ and V^4+^ are present in the EG/VO_2_ composites. The V^3+^ is attributed to the conversion of some V^4+^ ions to V^3+^ due to the excess of C_6_H_8_O_7_-H_2_O during the hydrothermal process, resulting in the production of trivalent vanadium. By drawing on relevant references [42,43], we have calculated the relative content of V^4+^ in elemental V to be 65%.

### 3.2. Electrochemical Testing

Cyclic voltammetry (CV) with a sweep rate of 1 mV/s was tested on an electrochemical workstation (AUTOLAB PGSTAT302N, Metrohm, Herisau, Switzerland). Electrochemical impedance (EIS) was tested on an electrochemical workstation (AUTOLAB PGSTAT302N, Metrohm, Herisau, Switzerland) at a frequency of 10^−2^~10^5^ Hz with the amplitude of 5 mV. Constant-current charging and discharging and multiplicity tests were performed on a battery test system (NEWARE, CT-4008, Shenzhen, China). All electrochemical tests were carried out in 1 mol/L ZnSO_4_ electrolyte in the voltage range of 0.2 to 1.0 V.

**Figure 5:**
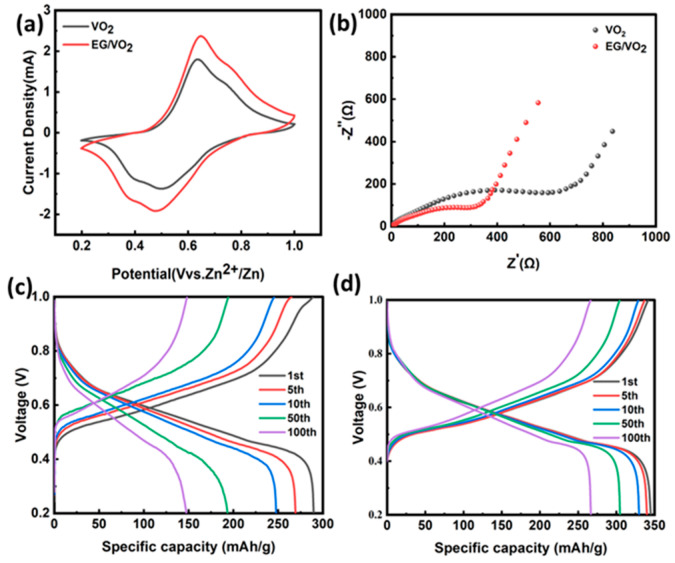
(**a**) CV curve of the VO_2_ and EG/VO_2_. (**b**) Electrochemical impedance spectra of the VO_2_ and EG/VO_2_. (**c**) The charge–discharge profiles of the VO_2_ battery at 0.1 A/g. (**d**) The charge–discharge profiles of the EG/VO_2_ battery at 0.1 A/g.

It can be observed from Figure 5a that the sample exhibits two pairs of redox peaks, indicating a multiple-step process of Zn^2+^ embedding and intercalation in the cathode material. Furthermore, the valence state of the vanadium element changes during this process. We think that the electrochemical behavior of VO_2_(B) in aqueous zinc-ion batteries is dominated by the embedding/de-embedding of Zn^2+^/[Zn(H_2_O)_6_]^2+^ in the electrolyte, while the embedding of Zn^2+^ induces the reduction of V. The V^4+^ supplied by the electrode material is gradually reduced to V^3+^ during the discharge process. Our analyses are consistent with the results of the related literature [44,45].

The oxidation peaks of the composites corresponded to the same potentials with good symmetry. Figure 5b shows a comparison of the electrode impedance of pure VO_2_ and EG/VO_2_ composites. The semicircle located in the high-frequency region corresponds to the charge transfer resistance, while in the low-frequency region, a straight line corresponds to the diffusion resistance of the electrolyte. Among them, the R_ct_ of the electrode material is the key factor affecting the electrochemical performance of the device. The R_ct_ values of pure VO_2_ and EG/VO_2_ materials are 639 and 356 Ω, respectively, which indicates that the vanadium dioxide composite with expanded graphite possesses a smaller Faraday resistance, corresponding to its zinc ion diffusion coefficient, which is the largest in the charging and discharging process of the battery, and the composite material has a faster de-embedding rate of zinc ions, which makes it easier for zinc-ion batteries to diffuse the ions in the charging and discharging process. 

Figure 5c,d present the charge–discharge curves of pure VO_2_ and EG/VO_2_ at a 0.1 A g^−1^ current density under different numbers of charge–discharge turns. The two materials exhibit initial discharge-specific capacities of 289.7 and 345.0 mAh/g, respectively, with both demonstrating a first-cycle Coulombic efficiency of 100%. When the battery cycles to the 100th lap, the discharge-specific capacity is 149.6 and 267.9 mAh/g, and the Coulombic efficiency is 99.4% and 100%, respectively. After 100 charging and discharging cycles, the charging and discharging curves of the battery are still stable, which indicates that the composites have stable zinc storage reversibility. A comparison of the discharge-specific capacity of the battery reveals that the capacity of the expanded graphite composite is significantly higher than that of the pure vanadium dioxide material, and the capacity retention and coulombic efficiency of the composite material show obvious advantages compared with that of the VO_2_ composite without expanded graphite. This result indicates that the inclusion of expanded graphite not only provides excellent conductivity of the electrode material but also stabilizes the crystal structure of vanadium dioxide. Expanded graphite’s good electrical conductivity and layered structure provide the material with a good conductive pathway and also a stable skeletal structure. These properties accelerate the electrochemical de-embedding of zinc ions on the composites, resulting in higher reversible specific capacity and faster embedding/de-embedding capability. The composites have well-developed pores of various pore sizes, and this multistage and interpenetrating pore structure is very favorable for ion diffusion and transport.

**Figure 6:**
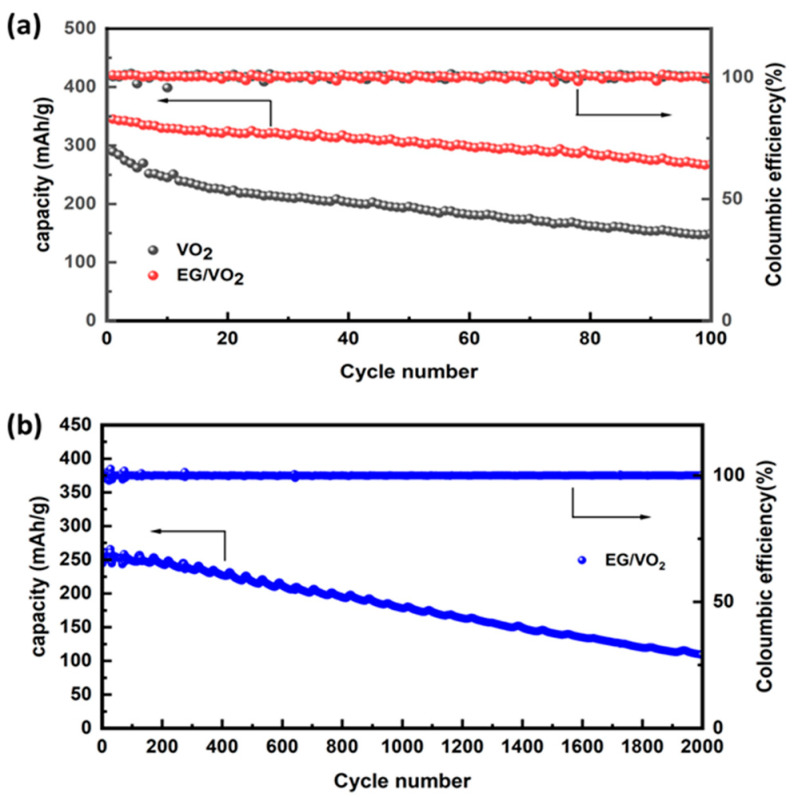
(**a**) Cycle diagram of two materials. (**b**) Long cycle diagram of EG/VO_2_ in 2 A/g.

Figure 6a demonstrates the cycling performance of the material at a current density of 0.1 A g^−1^. It can be more intuitively seen that the composite material exhibits excellent cycling stability, with a retention rate of about 80% and a Coulombic efficiency of more than 99% after 100 cycles, despite a slight decrease in capacity, which suggests that the composite material possesses a more excellent electrochemical performance. This advantage is attributed to the material’s abundance of active sites, which enables the zinc ions to carry out the de-embedding reaction in a stable manner. At the same time, the high crystallinity also ensures that the material has good stability in aqueous electrolytes, which is in line with the results of previous analyses. We investigated the long-cycle charge–discharge performance of EG/VO_2_ at a high current density of 2 A/g. The experimental results are shown in Figure 6b, where the Coulombic efficiency experienced fluctuations at the beginning of the cycle and then stabilized at 100% in the subsequent cycles. This phenomenon may be attributed to the partial dissolution of the material during the initial high-current-density charging and discharging of the battery. As a result, some zinc ions embedded in the material may not detach into the electrolyte through electrode reactions, leading to a slight fluctuation in Coulombic efficiency. However, as easily soluble components are reduced, the charging and discharging efficiency is successfully maintained at 100%. This also indicates that the structure of the composite material did not undergo significant damage. From the figure, it can be observed that the discharge-specific capacity of the material maintained a relatively stable state from 0 to 200 turns, and the specific capacity was 108.9 mAh/g after 2000 turns of cycling, which was maintained by 41%, indicating that it has excellent high-current long-cycle stability. Under the condition of high current density, the discharge-specific capacity of the electrode also showed high specific capacity although there was a certain degree of attenuation, which proved that the composite electrode had good reversibility and stability of charge and discharge. This conclusion is also confirmed in the relevant literature by non-in situ XRD [44].

**Figure 7:**
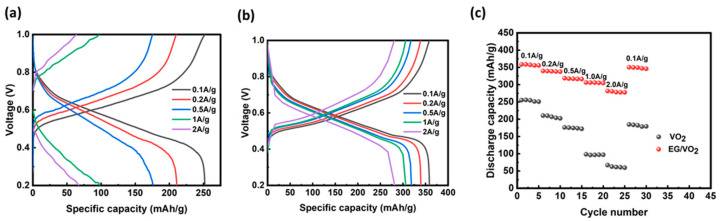
(**a**) The charge–discharge profiles of VO_2_ at different current densities. (**b**) The charge–discharge profiles of EG/VO_2_ at different current densities. (**c**) Multiplicity plots for two materials.

The charge–discharge curves of pure VO_2_ and EG/VO_2_ at different current densities are shown in Figure 7a,b, and the shape of the charge–discharge curves of the composites as shown in the figure received almost no influence from the increase in current density, but the plateau of the discharge curves disappeared and the curves fluctuated considerably when the current density of pure VO_2_ was increased to 1 A g^−1^ and 2 A g^−1^, indicating that the high current had caused the VO_2_ material, which was not protected by the EG, some degree of negative impact, and the change in the discharge curve corresponds to its dismal capacity retention under high current. In conclusion, the composites still show better multiplicity performance under different current densities. 

The results of the multiplication performance test are shown in Figure 7c, during which the current density was set to 0.1 A/g, 0.2 A/g, 0.5 A/g, 1 A/g, 2 A/g, and finally returned to 0.1 A/g. The multiplication performance is the ability of the battery to store or release more electrical energy in a shorter period, so with the increase in the charging and discharging current densities, the degree of the battery’s specific capacity retention becomes the key to measure the superiority of its multiplicity performance. For EG/VO_2_ materials, the electric capacity exhibits a certain degree of decrease with the increase in current density. This is due to the fact that as the charge–discharge current density increases, the electron transfer rate inside the battery increases dramatically, while the charge–discharge time is shortened. However, for the ion de-embedding process in the electrode material, the electrochemical reaction speed cannot keep up with the electron transfer frequency, leading to an early end of the electrode reaction, which results in a decrease in the specific capacity value. It can be visualized in the figure that the multiplicative performance of pure vanadium dioxide as an electrode material is very different from that of the expanded graphite composite, and when the current density is increased to 2 A g^−1^, the discharge-specific capacities exhibited by the pure VO_2_ and EG/VO_2_ working electrodes are 67.1 and 277.8 mAh/g, respectively, and the capacity retention rate is 26% concerning that under the current of 0.1 A g^−1^ and 78%, respectively, so the composites exhibit higher capacity release capability even at the same high current state and under the same electrolyte environment, thus showing excellent multiplicative performance. When the current density was restored to 0.1 A g^−1^, the respective discharge-specific capacities of the materials were 184.6 and 350.4 mAh/g, and the capacity retention rates were 72% and 98%, respectively, relative to those at 0.1 A g^−1^ current density. This indicates that the composites still exhibit excellent stability under the same experimental conditions, even after high current density shocks. 

## 4. Conclusions

Aqueous zinc-ion batteries are emerging as a very promising technology for large-scale energy storage batteries due to the high safety, low cost, and abundant material resources. However, its successful application in commercial production is still limited by the rate capability and cycle life of the cathode material. In this paper, EG/VO_2_ composites were prepared by a hydrothermal method, and the successful synthesis of the composites was determined by characterization means such as SEM and XPS. The electrode materials composited with expanded graphite then showed more excellent capacity, cycling performance, and multiplicity performance when compared with aqueous zinc-ion batteries assembled with single VO_2_ under the same conditions. Comparison of anode material performance are shown in Table 1. The material possesses a high discharge-specific capacity of 345 mAh g^−1^ at 0.1 A g^−1^ with a Coulombic efficiency close to 100%, a capacity retention of 77% after 100 cycles, and it exhibits a discharge-specific capacity of 277.8 mAh g^−1^ with a capacity retention of 78% when the current density is increased by 20 times. It was also subjected to a long cycle test. The data proved that the composites effectively improved the poor cycling performance of vanadium-based materials, which had been criticized, and the composites maintained a stable Coulombic efficiency of 100% at a high current density of 2 A/g and still maintained a specific capacity of 108.9 mAh/g after 2000-turn cycling, and the considerable electrochemical performance is of significance for the application of vanadium-based materials in aqueous zinc-ion batteries.

## Figures and Tables

**Table 1:** Comparison of anode material performance.

Material	Current Density	Discharge-Specific Capacity	Reference
V_2_O_5_	0.2 A g^−1^	470 mAh g^−1^	[20]
V_2_O_5_/(VOG)	0.3 A g^−1^	144 Wh kg^−1^	[21]
Al_0.2_V_2_O	0.1 A g^−1^	448.4 mAh g^−1^	[22]
VO_2_(B)	0.05 A g^−1^	357 mAh g^−1^	[23]
(Ni)VO_2_	5 A g^−1^	182 mAh g^−1^	[24]
V_2_O_3_@C	2 A g^−1^	853 mAh g^−1^	[25]
V_3_O_7_·H_2_O	1 C	375 mAh g^−1^	[26]
Ni_x_V_6−x_O_13_	1 A g^−1^	302.6 mAh g^−1^	[27]
VO_2_	0.1 A g^−1^	289.7 mAh g^−1^	This text
EG/VO_2_	0.1 A g^−1^	345 mAh g^−1^	This text

## Data Availability

Data are contained within the article.
